# Gli2a protein localization reveals a role for Iguana/DZIP1 in primary ciliogenesis and a dependence of Hedgehog signal transduction on primary cilia in the zebrafish

**DOI:** 10.1186/1741-7007-8-65

**Published:** 2010-04-19

**Authors:** Hyejeong Rosemary Kim, Joanna Richardson, Freek van Eeden, Philip W Ingham

**Affiliations:** 1 MRC Centre for Developmental & Biomedical Genetics, University of Sheffield, Sheffield S10 2TN, UK; 2 Current Address: MRC Centre for Developmental Neurobiology, New Hunt's House, Guy's Campus, London SE1 1UL, UK; 3 Current Address: Institute of Molecular and Cell Biology, Proteos, 61 Biopolis Drive, Singapore 138673

## Abstract

**Background:**

In mammalian cells, the integrity of the primary cilium is critical for proper regulation of the Hedgehog (Hh) signal transduction pathway. Whether or not this dependence on the primary cilium is a universal feature of vertebrate Hedgehog signalling has remained contentious due, in part, to the apparent divergence of the intracellular transduction pathway between mammals and teleost fish.

**Results:**

Here, using a functional Gli2-GFP fusion protein, we show that, as in mammals, the Gli2 transcription factor localizes to the primary cilia of cells in the zebrafish embryo and that this localization is modulated by the activity of the Hh pathway. Moreover, we show that the Igu/DZIP1protein, previously implicated in the modulation of Gli activity in zebrafish, also localizes to the primary cilium and is required for its proper formation.

**Conclusion:**

Our findings demonstrate a conserved role of the primary cilium in mediating Hedgehog signalling activity across the vertebrate phylum and validate the use of the zebrafish as a representative model for the *in vivo *analysis of vertebrate Hedgehog signalling.

## Background

Signalling by Hedgehog (Hh) family proteins plays a key role in the development of many animal species, controlling both cell fate specification and cell proliferation in a variety of contexts. In addition, Hh signalling is deployed to regulate tissue homeostasis post-embryonically and aberrant activity of the Hh pathway has been implicated in a number of cancers [1,2]

Most of the components of the Hh signal transduction pathway, including the transmembrane proteins Patched (Ptc) and Smoothened (Smo), the Gli family transcription factor Cubitus interruptus (Ci) and the kinesin-like family protein Cos-2, were first identified by genetic analysis in *Drosophila*, [3]. Cos-2 binds to Ci and recruits a number of protein kinases including Fused, a positive regulator and Protein kinase A (PKA), a negative regulator of the pathway [4,5]. PKA phosphorylates Ci, priming it for proteolytic cleavage to yield a truncated form that acts as a transcriptional repressor of Hh target genes. Activation of the pathway disrupts the Cos-2 complex [6], leading to the accumulation of the full length, activating form of Ci, which enters the nucleus and activates transcription of Hh target genes [3].

Although the roles of the Ptc, Smo and Gli proteins in Hh signalling appear to have been highly conserved through evolution, several lines of evidence have pointed to a divergence of the intracellular signalling mechanism between flies and vertebrates. In particular, targeted mutation of the murine orthologue of Fused revealed that it is dispensable for Hh signalling in mice [7,8], while manipulation of the activity of two murine homologues of Cos-2, Kif7 and Kif27, implied that they are similarly not involved in the pathway [9]. At the same time, the discovery that mutations in a number of genes encoding IFT proteins disrupt Hh signalling in the mouse, implicated the primary cilium as a key cellular compartment for Hh signal transduction and led to the suggestion that this unique organelle has subsumed the role of Cos-2 in organizing the intracellular pathway components, at least in mammalian cells [10]. Consistent with this, green fluorescent protein (GFP) tagged forms of the Gli proteins have been shown to localize to the tips of the primary cilia when expressed in primary cultures of mouse limb bud cells [11].

In the zebrafish, however, analyses based on morpholino mediated knock-down of gene activity indicated that both Fused and Kif7 are required for Hh signalling [12,13]. This suggests that the pathway in zebrafish is more similar to that in *Drosophila *and raises doubts about the involvement of the primary cilium in Hh signalling throughout the vertebrates. Moreover, although mutations that disrupt the localization of Smo to the cilium have been shown to cause a loss of Hh pathway activity in zebrafish embryos [14,15], zebrafish mutant for three of the intraflagellar transport (IFT) protein encoding genes have been reported to show no detectable affect on the activity of the pathway [16].

Mouse IFT mutants have paradoxical phenotypes suggesting both a partial gain and partial loss of Hh function in different organs. Thus, the *IFT88/polaris *, *IFT57/Hippi *and *IFT172/wimpole *mutants all exhibit polydactyly, indicative of an increase in Hh pathway activity, while at the same time manifesting a loss of Shh-dependent neurons in the neural tube [17,18]. One way of rationalizing these opposing effects is to posit an overall reduction in the levels of Gli proteins in the mutants, the different consequences reflecting the differing contributions of the activator (Gli-A) and repressor (Gli-R) forms in different tissues. Thus, in the limb bud, where Shh acts principally to de-repress target genes by lowering Gli-R levels, a diminution of Gli-R should lead to a gain of function phenotype. However, in the neural tube, where Shh acts principally to activate target gene transcription by enhancing Gli-A levels, any reduction in these levels should result in a loss of function phenotype.

Zebrafish embryos homozygous for mutant alleles of the *iguana *(*igu*) locus exhibit a similarly paradoxical Hh phenotype. In this case they manifest as a gain of pathway activity in the myotome and a loss of pathway activity in the neural tube [19,20]. Based on an analysis of the *igu *phenotype and the apparent sub-cellular distribution of a GFP-Igu fusion protein, it was proposed that Igu regulates the nucleo-cytoplasmic shuttling of Gli-A and Gli-R proteins in some manner [19,20].

In order to investigate the role of Iguana/DZIP1 further, we have generated a transgene encoding a GFP-tagged form of the Gli2a protein and used this to analyse its sub-cellular distribution in wild-type and mutant embryos. We find that, as in mammalian cells, Gli2a localizes to the primary cilia of cells in zebrafish embryos and that this localization is modulated in response to Hh signalling. Analysis of Gli2a localization reveals a prominent defect in primary cilia in *igu *mutant embryos, implicating Igu/DZIP1 function in primary ciliogenesis. Taken together, our data suggest that the role of the primary cilium as a centre for processing Gli proteins in response to Hh signalling is conserved throughout the vertebrates.

## Results

### A functional GFP-tagged form of Gli2a localizes to the primary cilium in zebrafish embryos

**Figure 1:**
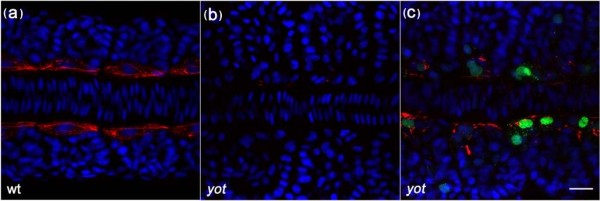
**A Gli2a-GFP fusion protein is capable of rescuing adaxial F59 expression in *you-too *(*yot*) mutant embryos**. Newly fertilised eggs from a *yot/+* incross were injected with modified BAC DNA containing GFP tagged Gli2a. The injected embryos were fixed at the 12 somite stage and labelled with anti-F59 (red) in order to detect slow lineage muscle fibres (a), the expression of which is eliminated in *yot *mutant embryos (b). F59 expression was restored specifically in the Gli2a-GFP expressing cells of transient transgenic mutant embryos (c), indicating that GFP tagged Gli2a protein is functional. Dorsal view, anterior to the left. Nuclei: DAPI in blue. Scale bar: 20 μm

A key process in the transduction of the Hh signal in all organisms is the regulation of the cleavage and nuclear accumulation of the Gli transcription factors. A major obstacle to the analysis of this process in the zebrafish has been the paucity of reagents with which to detect the various forms of the different Gli proteins. In order partially to circumvent this limitation, we developed a GFP-tagged form of the zebrafish Gli2a protein, which is encoded by the gene inactivated by the *yot *mutation [21]. We identified a BAC containing the entire Gli2a transcription unit and flanking 5' and 3' sequences and used recombineering [22] to insert the GFP coding sequence in frame at the C-terminus end of the Gli2a open reading frame such that the full length (Gli-A) form of the protein, but not the truncated (Gli-R) form, should be tagged with GFP. In order to test the function of this tagged protein, we injected the modified BAC into *yot *mutant embryos. The ability of the tagged protein to rescue the effects caused by mutation of the endogenous gene was assayed by staining the embryos with the mAb F59 in order to detect slow type muscle fibres in the developing myotome. In wild type embryos, F59 accumulates specifically in adaxial cells in response to Hh signalling (Figure 1a) [23,24] and this response is blocked in *yot *homozygous embryos which are therefore devoid of adaxial F59 expression (Figure 1b). We found that F59 expression is restored in injected *yot *mutant embryos, specifically in cells expressing the GFP-tagged protein (Figure 1c). Thus, we conclude that the full-length GFP-tagged Gli2a protein is functional.

**Figure 2:**
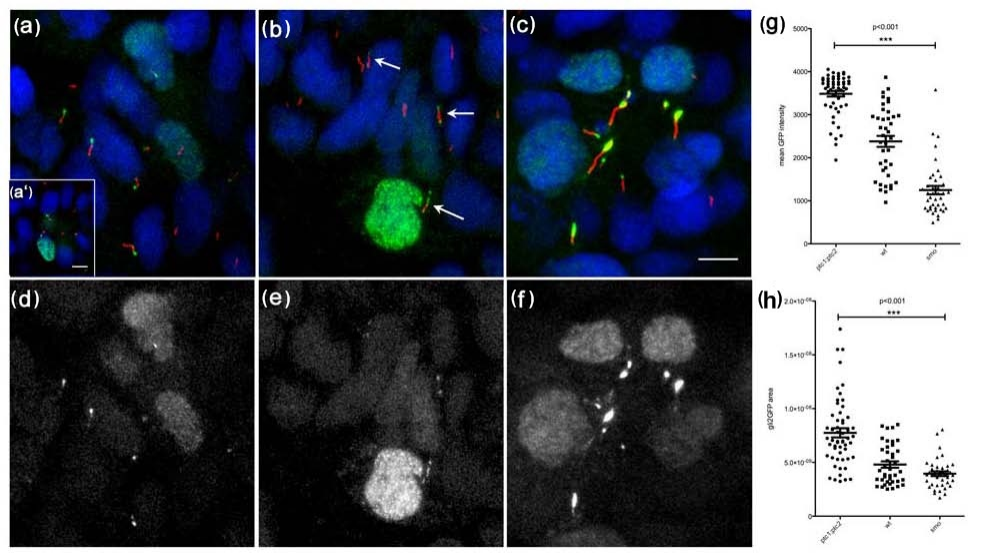
**Gli2a-GFP localizes to the distal tips of primary cilia in paraxial mesodermal cells (18 somite stage) and its localization is modulated by the activity of Hedgehog signalling**. Gli2a-GFP injected embryos were labelled with mAb acetylated tubulin (red in a, b and c) and gamma tubulin (red in a'). This revealed that Gli2a-GFP was localized at the distal tip of primary cilia (a) and that its expression was excluded from the basal bodies (a'). In *smoothened *(*smo*) mutant embryos, levels of Gli2a-GFP were diminished (b) and the signal was dispersed along the cilia or localized both at the distal tip and at the basal bodies (arrows). In *ptc1:ptc2 *double mutant embryos, by contrast, high levels of Gli2a-GFP accumulated at the distal tip of the cilia (c). Panels (d-f) show the green channel images of (a-c), respectively. The intensity and the area of Gli2a-GFP at the tip of the cilia were measured in wild type and mutant embryos and the difference between the three groups was analysed by one way ANOVA test. This revealed a significant difference in protein levels between wild type, *smo *and *ptc1;ptc2 *mutant groups with *P < 0.001 *(g and h). Scale bars: 5 μm

As well as the anticipated accumulation of the Gli2a-GFP fusion protein in nuclei, we also observed discrete puncta of GFP signal in expressing cells. A closer examination suggested these to be associated with primary cilia. In order to confirm this, we double stained transiently transformed embryos with antibodies specific for acetylated tubulin or γ-tubulin which label the axonemes and basal bodies of primary cilia, respectively. This showed that the Gli2a-GFP protein was excluded from the basal bodies (Figure 2a') but localized to the distal tip of the primary cilia (Figure 2a). In order to exclude the possibility that this localization is mediated by the GFP moiety in the fusion protein, we analysed the distribution of GFP expressed in transiently transgenic zebrafish embryos and found no evidence of its localization to primary cilia (Additional file 1, Supplementary Figure 1.)

As a result of the mosaic nature of the transgene expression, we analysed its expression in individual cells in a number of embryos and pooled the data. In a sample of 87 Gli2a-GFP expressing cells, we found that the fusion protein localized to the distal tip of the primary cilia in the majority (64%) of cases. In about one third of these (29/87) the protein also localized to the nucleus with varying levels of intensity, but only in rare cases (2/87) was there signal in the cytoplasm in addition to the tip of the cilia. In a second sample of 83 cells showing ciliary localization of the fusion protein, we found 15 cases with low-level expression at the base of the primary cilia in addition to strong expression at the tip.

### The localization of Gli2a-GFP to primary cilia is modulated by Hh pathway activity

In order to investigate whether the cilial localization of Gli2a is modulated by Hh pathway activity, we next analysed the distribution of the Gli2a-GFP fusion protein in Hh pathway mutant embryos transiently transgenic for *TgBAC(gli2a-GFP)*. Ptc proteins act both as receptors for the Hh ligand and as repressors of the Hh signal transduction pathway: in the absence of Hh, Ptc inhibits the activity of the signal transducer Smo which, in turn, controls the intracellular balance of Gli-A and Gli-R. Thus, the loss of Ptc function leads to a de-repression of Smo activity and a concomitant increase in Gli-A forms. In embryos doubly homozygous for the *ptc1 *and *ptc2 *loss of function alleles [25], the Gli2a-GFP protein mostly remained localized to the tip of the cilia but showed a higher signal intensity than in wild-type siblings (Figure 2c); Gli2a-GFP was expressed only at the tip of the cilia in most of the cilia examined (74/83) while, in a minority of cases (9/83), diffuse signal was distributed throughout the cilia. In contrast, in homozygous *smo* mutant embryos [26,27], the levels of Gli2a-GFP were significantly diminished; in addition, the signal became dispersed along the axoneme or accumulated at both the distal tip and the basal body of the cilium (42/51; Figure 2b). We measured the intensity of the Gli2a-GFP expression at the tip of the cilia in wild type, *ptc1;ptc2 *mutant and *smo *mutant embryos and compared its intensity among the three groups. There was a significant difference in the intensity between wild type, *smo *and *ptc1;ptc2 *double mutants (Figure 2g-2h). Taken together, these data indicate that the localization of Gli2a to primary cilia is modulated by Hh pathway activity and suggest that, as in mammals, the intracellular transduction of the signal is mediated via this organelle. Interestingly, there was also a significant difference in the length of primary cilia in which Gli2a-GFP was localized compared to those which were unlabelled and between primary cilia in *ptc1;ptc2 *double mutant embryos compared to those of wild-type embryos (Additional file 2: Supplementary Figure 2).

### Gli2a-GFP localization reveals defective primary cilia in *iguana *mutant zebrafish embryos

**Figure 3:**
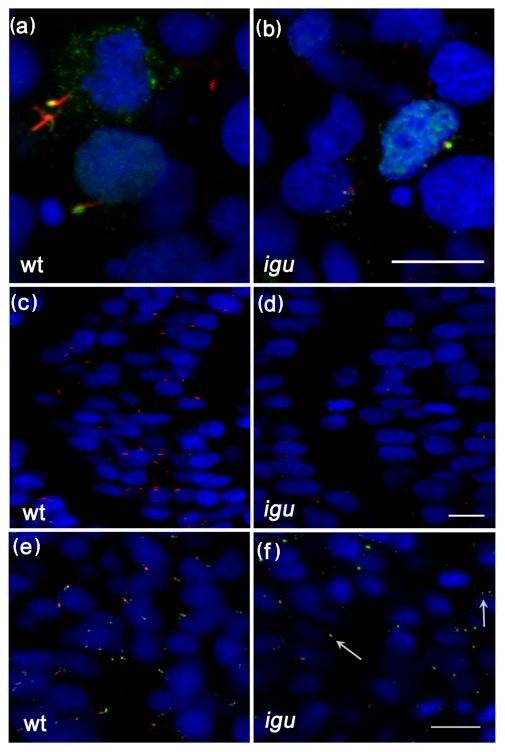
**Primary cilia are truncated or missing from somitic cells in *Iguana (igu) *mutant embryos**. Gli2a-GFP co-localized with acetylated tubulin (red) in *igu *mutant embryos in presumptive axonemes (b) that were severely truncated relative to those of wild type primary cilia (a). Truncated cilia (red) were only occasionally present in *igu *embryos (d) in contrast to wild type (c). Basal bodies (green: γ-tubulin in e, f) were present in all cells in *igu *mutant embryos (f) as in wild type (e) and were associated with the truncated axonemes (arrows). Scale bars: 10 μm

Previous analyses of the *igu *mutant phenotype led to the suggestion that the DZIP protein encoded by the mutated gene regulates the nuclear-cytoplasmic trafficking of the Gli proteins in response to Hh signalling [20]. In order to investigate this proposal further, we generated *igu *mutant embryos transiently transgenic for *TgBAC(gli2a-GFP) *and analysed the sub-cellular distribution of the fusion protein. As in wild type siblings, GFP signal could be detected in the nuclei of the *igu *mutant embryos and also, occasionally, in discreet puncta associated with expressing cells. Staining with anti-acetylated tubulin revealed a co-localization with these puncta. However, in contrast to the elongated axonemes typical of those in wild type embryos, the acetylated tubulin and Gli2a-GFP appeared to coalesce in short stumpy structures that we take to represent vestigial primary cilia (Figure 3b). Consistent with this interpretation, double staining with anti-γ-tubulin revealed these to be associated with basal bodies. However, whereas basal bodies were present in all cells in *igu *mutant embryos, as in wild type, the incidence of axoneme-like structures was greatly reduced (Figure 3f).

### Iguana/DZIP1 is required for primary but not motile cilliogenesis

In contrast to the primary cilia that are present on most cells and have a '9+0' microtubule organization, motile cilia are characterized by a '9+2' microtubule organization, are usually longer than primary cilia and are found only on certain specialized cell types. In zebrafish, motile cilia are present in the pronephros, ventral canal of the spinal cord, the brain and in Kupffer's vesicle where their beating facilitates fluid flow. The loss of these motile cilia results in kidney cysts, hydrocephalus, and left-right asymmetry defects [28]. Motile cilia are also present in the otic vesicle and olfactory sensory neuron [29,30]. The role of motile kinocilia in the formation of otolith was reported in zebrafish [29], while in mammals, non-motile olfactory cilia are necessary for detection of the odorants in the environment [31].

**Figure 4:**
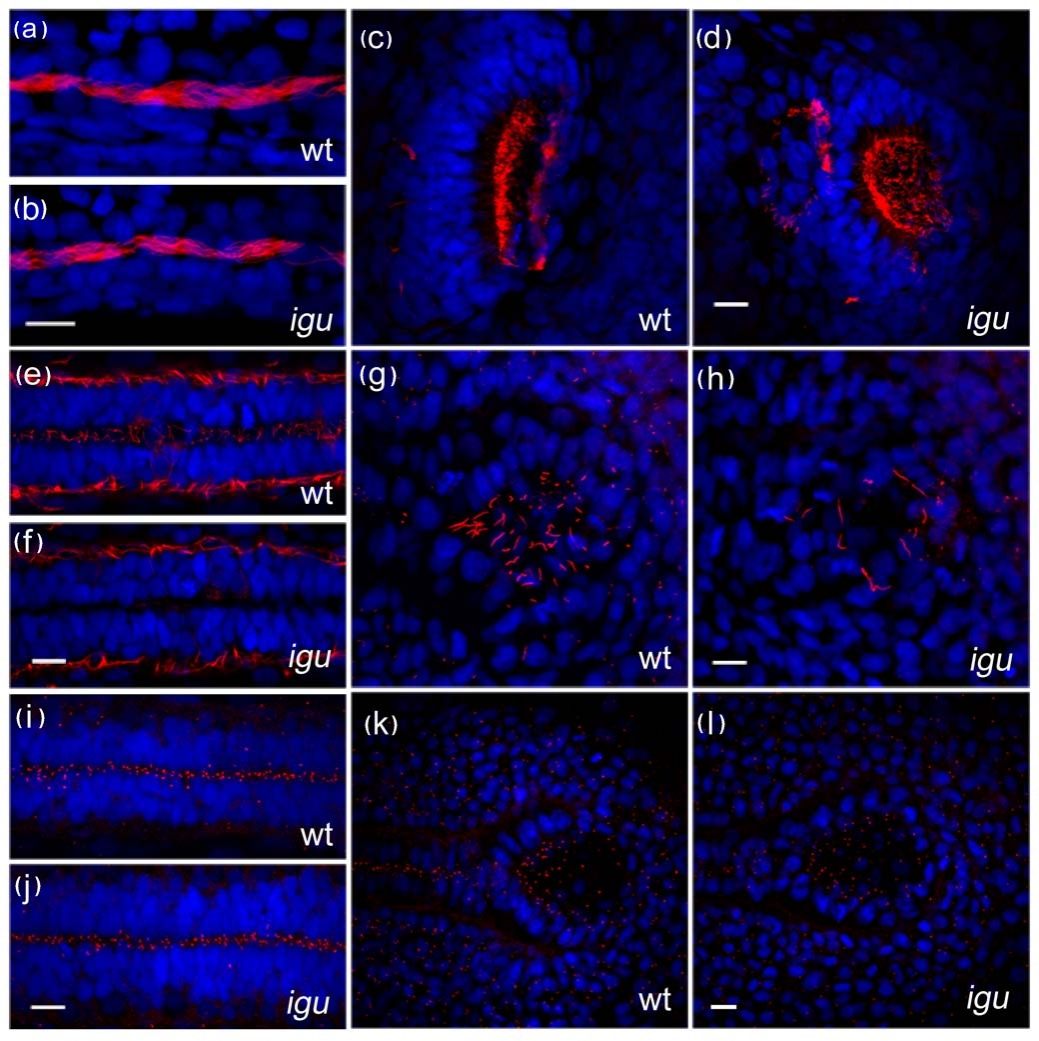
**Motile cilia are largely unaffected by the loss of Iguana (Igu) function**. Motile cilia (red: acetylated tubulin in a-h) in the pronephros at 48 hpf in *igu *mutant embryos appeared normal (b), as did those in the olfactory pits (d). By contrast motile cilia were largely absent from the floorplate at 28 hpf (f) and those in Kupffer's vesicle were significantly reduced in number (h) in 10 somite stage *igu *mutant embryos. Basal bodies (red: γ-tubulin in i-l) in the *igu *mutants, by contrast, were formed normally at all stages examined in the floorplate (j) and Kupffer's vesicle (l). Scale bars: 10 μm

**Figure 5:**
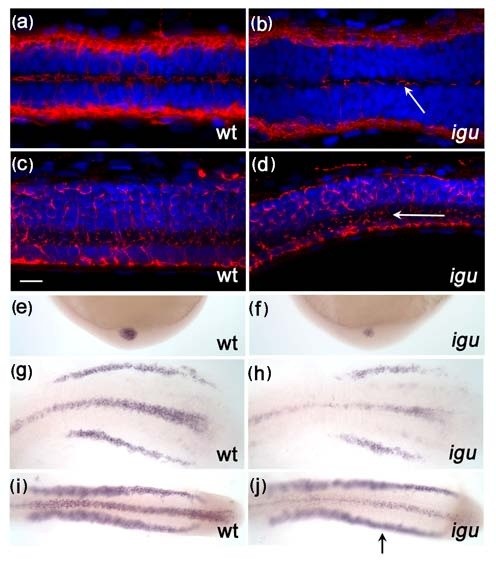
**Motile cilia in the floorplate recover in *Iguana (igu) *mutant by 5 dpf**. A few motile cilia (red: acetylated tubulin) were apparent in the floorplate of *igu *mutants at 48 hpf (b, arrow), and were almost completely recovered by 5 dpf (d, arrow). *In situ *hybridization reveals that *foxj1a *expression is down-regulated in Kupffer's vesicle (f) and the floor plate (h, j) at the end of gastrulation and early somitogenesis (h:8 somite, j:14somite), respectively. In contrast, expression in the pronephros was normal (j, arrow). Scale bar: 10 μm. Dorsal view: a-b & g-j, lateral view: c-f

**Figure 6:**
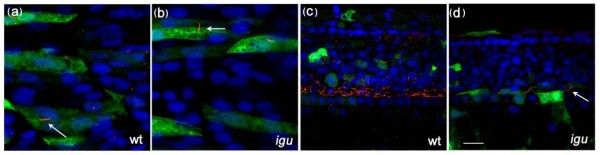
**Ectopic expression of *foxj1a *in somites results in ectopic motile cilia formation in both wild-type and mutant embryos and rescues motile cilia formation in the floorplate of *Iguana (igu) *mutant embryos**. Ectopic motile cilia were induced in somitic cells expressing *foxj1a *(marked by green fluorescent protein expression) both in wild type and *igu *mutant embryos (a and b, arrows). Motile cilia were restored in the floorplate of *igu *mutant embryos in cells expressing *foxj1a *(d, arrow). Scale bar: 10 μm

In order to establish whether Igu function is required generally for all cilliogenesis, we examined two prominently ciliated structures, the pronephros and olfactory pits, in *igu *mutant embryos at 48 hpf and 60 hpf, respectively. In contrast to the truncated primary cilia observed in paraxial mesodermal cells, the motile cilia in the pronephros and olfactory pits appeared to be formed normally in *igu *mutant embryos (Figure 4b and 4d), although the morphology of the olfactory pits seemed to be somewhat affected. In contrast, motile cilia were absent from the floorplate of *igu *mutant embryos at 28 hpf (Figure 4f) while those located in Kupffer's vesicle were reduced in number in *igu *mutant embryos at 10 somite stage (Figure 4h). However, in both cases, the distribution of basal bodies appeared relatively normal (Figure.4i, j, k and 4l). At 48 hpf, however, a few motile cilia were detected in the floorplate of *igu *mutants (Figure 5b) and by 5 dpf their formation was almost completely recovered (Figure 5c and 5d). Differential interference contrast imaging of the tail region of the floor plate of live *igu *mutant embryos revealed that these cilia were indeed motile (data not shown). These findings suggest that DZIP1/Igu function is required specifically for the formation of primary cilia and that it is not essential for motile cilia formation. The transcription factor Foxj1a has been shown to be both necessary and sufficient for motile cilia formation and transcription of the *foxj1a *gene in the early zebrafish embryo is regulated by Hh signalling [32]. The reduction of motile cilia in Kupffer's vesicle and their delayed formation in the floorplate of *igu *mutant embryos might thus be an indirect consequence of the attenuation of Hh signalling in these structures. Consistent with this interpretation, we found that in contrast to the situation in wild-type embryos, the levels of *foxj1a *expression in Kupffer's vesicle at the end of gastrulation (Figure 5e and 5f) and in the floorplate before 24 hpf (Figure 5g-j) were reduced relative to those in the pronephros. In order to test the inference that this reduced expression underlies the delay in motile cilium differentiation, we used a heat shock inducible form of *foxj1a *to drive its expression in *igu *mutant embryos. Such expression effectively rescued the differentiation of motile cilia in the floorplate of *igu *mutants (Figure 6d) and also induced ectopic cilia in myotomal cells (Figure 6b), as has previously been described to occur in wild type embryos [32].

### The Iguana/DZIP1 protein localizes to nuclei and to the basal body of primary cilia

**Figure 7:**
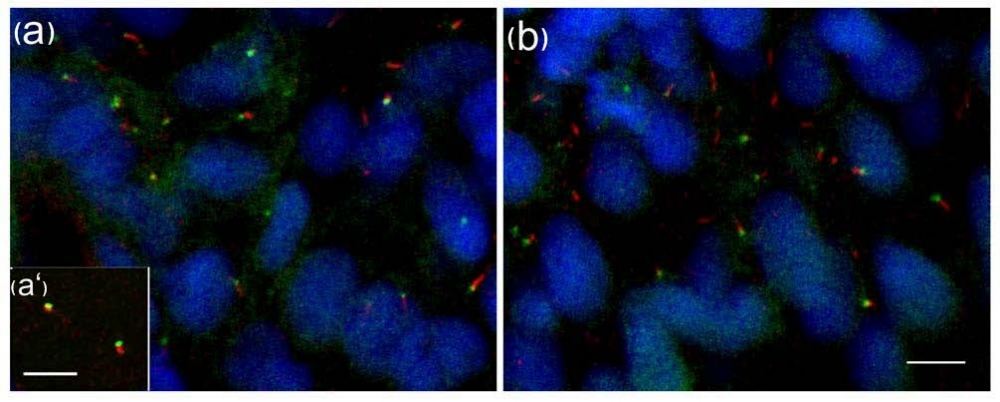
**GFP-tagged Iguana (Igu) localizes to basal bodies of primary cilia in zebrafish embryos**. *GFP-igu *mRNA was injected into 1-cell stage embryos and injected embryos fixed and labelled with anti-acetylated tubulin (red). Punctate GFP-Igu localization was excluded from the axoneme (a). Co-injection of mRNA encoding tdTomato fused GTAP3 (γ-tubulin associated protein 3) and GFP-Igu, revealed co-localization of both proteins at the basal bodies in live early somitogenesis stage embryos (a'). The basal body localization of GFP-Igu was un-altered in embryos expressing dnPKA (b). Scale bars: 5 μm

In an earlier study, the sub-cellular distribution of the Igu/DZIP1 protein was analysed using a GFP fusion protein encoded by an mRNA injected into newly fertilised embryos [20]. Following fixation of injected embryos, the protein was found to localize predominantly to the cytoplasm. However, co-injection of an mRNA encoding a dominant negative form of the PKA regulatory sub-unit (dnPKA; which activates Hh target gene expression by abrogating proteolytic processing of the full length forms of Gli proteins), was reported to induce a re-localisation of the fusion protein to the nucleus [20]. In the light of our new findings, we repeated these experiments, specifically to address the relationship between Igu/DZIP1 protein localization and its role in ciliogenesis. Inspection of the sequence of the cDNA clone encoding the GFP-Igu fusion protein revealed three nucleotide substitutions that cause a divergence from the published wild type amino acid sequence at three residues. We corrected these substitutions by *in vitro *mutagenesis (see Materials and Methods) and injected the modified mRNA into embryos derived from *igu *heterozygous parents. Homozygous mutant embryos thus injected showed a complete rescue to wild type morphology (confirmed by genotyping the injected mutant embryos). We observed discrete puncta of GFP signal in each cell, which we surmised represented localization to the primary cilia. In order to confirm this, we fixed injected embryos and stained them with anti-acetylated tubulin and γ-tubulin. This revealed that Igu protein is excluded from the axoneme (Figure 7a) and co-localizes with basal bodies (data not shown). We also visualised this co-localization in live embryos by simultaneous injection of mRNA encoding the GFP-Igu fusion and a γ-tubulin associated protein 3-tdTomato fusion (Figure 7a'). Co-injection of mRNA encoding dnPKA with the *GFP-igu *mRNA had no effect on cilia localization of the protein (Figure 7b).

## Discussion

Previous studies have implied a divergence in the mechanisms of Hh signalling between mammals and zebrafish [9], raising doubts about the role of the primary cilium in Hh signalling in the latter [10], a view reinforced by the apparent lack of effect of zebrafish IFT mutants on Hh signalling [16]. The recent finding that the role of Kif7 as a regulator of the Hh signal transduction is, in fact, conserved in mice [33-35] has, however, removed a major line of evidence in support of the case for divergence. Our data, together with complementary findings reported recently by Huang and Schier [36] now provide compelling evidence that Hh signals are indeed transduced in zebrafish, as in mammals, via the primary cilium. Here, we demonstrate that a functional Gli2a-GFP fusion protein localizes to the primary cilia of cells in the zebrafish embryo and that this localization is modulated by the activity of the Hh pathway. These findings are consistent with previous reports that Gli2 localizes to the tips of cilia in mouse limb-bud derived embryonic fibroblasts [11] and provide the first *in vivo *evidence for the role of Ptc and Smo in mediating such localization. Moreover, we show that loss of Igu/DZIP1 function, which has previously been demonstrated to cause a de-regulation of Gli activity in zebrafish embryos [19,20], disrupts the structure of primary cilia. We note that three other papers implicating DZIP1/Igu in primary ciliogenesis have been published since submission of this manuscript [37-39]. In line with these findings, complete ablation of the primary cilia by removal of both zygotic and maternal expression of oval/IFT88 clearly disrupts Hh signalling in the zebrafish embryo, leading to phenotypic effects remarkably similar to those of *igu *mutants [36].

By introducing a transgene encoding a Gli2a-GFP fusion protein into mutant embryos, we found that accumulation of the protein at the tip of the primary cilium is promoted by Hh pathway activity. Thus, in embryos doubly mutant for the genes encoding the Hh receptors Ptc1 and Ptc2, the levels of the fusion protein at the tip of the cilia are intensified, whereas in Smo mutants the levels were reduced and the protein was found in the basal bodies as well as along the axoneme. This suggests that in the absence of Hh signalling Gli2 is shuttled between the tip of the cilium and the basal body: the latter is known to be enriched for proteasomes [40] so may represent the site of processing Gli proteins to their repressor forms. Activation of the pathway results in a localization of the full-length protein at the tip of the cilium, thus removing it from proximity to the processing machinery. The increased length of primary cilia that accumulate Gli2-GFP at their tips or that a lack of functional Ptc1 and Ptc2 proteins was unexpected. However, recent studies have suggested that cilia length is modulated by cAMP levels in response to external stimuli [41,42]. Our findings provide a hint that Hh signalling may similarly influence the length of primary cilia; exactly how this is effected awaits further investigation.

Epistasis analyses of the *igu *mutation have previously demonstrated that the induction of ectopic Engrailed-expressing medial fast fibres (MFFs) in the embryonic myotome of mutant embryos depends critically upon Gli1 activity, implying that the loss of DZIP1 function, and hence in the light of our current analysis, of primary cilia, leads to a relative increase in the activity of this transcription factor. Notably, *igu *mutants were also shown to be resistant to the effects of PKA inhibition [19,20], which in wild type embryos abrogates the production of the repressor forms of the Gli proteins. The resistance of *igu *mutants to PKA inhibition implies that PKA does not have a significant impact on Gli activity in the absence of cilia. It follows that in *igu *mutants, levels of the Gli-R forms will be significantly reduced, leading to an increase in the relative levels of Gli-A forms. While such a shift in the Gli-A/Gli-R balance can account for the expansion of Hh dependent cell types in the myotome, it is notable that cell types dependent upon the highest levels of Hh signalling are reduced or absent from both the myotome and neural tube of *igu *mutants [19,20]. This implies that an additional cilia-dependent process is required for the maximal activity of Gli-A forms.

The localization of the GFP-Igu fusion protein to the basal body of the cilium is consistent with its role in ciliogenesis and mirrors that of IFT proteins [43] as well as of the chicken Talpid3 protein [44], mutation of which produces a phenotype strikingly similar to that of the zebrafish *igu *mutant [45]. Like Igu/DZIP1, Talpid3 and several IFT proteins are characterized by the presence of coiled-coil domains, a motif that mediates protein-protein interactions. This shared sub-cellular localization and structural motif suggests that Igu/DZIP1 may be a component of a multi-protein complex.

Although the primary cilia are severely truncated in *igu *mutant embryos, we find that motile cilia in the pronephric ducts, olfactory pits and neural tube are largely unaffected by the mutation. This implies a specific role for Igu/DZIP1 in primary ciliogenesis, which contrasts with the involvement of IFT proteins in the formation of both primary and motile cilia. We note, however, that some motile cilia are affected in *igu *mutants, namely those in the floorplate at earlier stages of embryogenesis and in the middle of Kupffer's vesicle. The latter have been implicated in generating the nodal flow that controls left right asymmetry in vertebrate embryos. Hence, their absence could account for the previous observation that L-R asymmetry is disrupted in *igu *mutant [46]. In the floor plate, motile cilia were observed only at the tip of the tail at 28 hpf. However, by 5 dpf, such cilia had recovered along the length of the neural tube. Our finding that the levels of expression of the gene encoding the Foxj1a transcription factor are reduced specifically in Kupffer's vesicle and floorplate cells in *igu *mutants is consistent with this effect on motile cilia being an indirect consequence of the attenuation of Hh signal transduction caused by the disruption of the primary cilia formation. That heat shock driven expression of Foxj1a can induce motile cilia formation in *igu *mutant embryos supports this view. The recovery of these cilia at later stage could reflect a gradual increase in the levels of Gli activity in *igu *mutants, as implied by the induction of ectopic MFFs in the myotome [19,20].

## Conclusion

Our findings provide further evidence for the role of the primary cilium in the modulation of Gli transcription factor activity in response to Hh signalling and confirm that such a role is conserved from teleosts to mammals. They also suggest a role for Hh signalling in controlling primary cilium length. The localization of the DZIP1 protein to the base of the primary cilium is consistent with its requirement for primary cilium assembly, as revealed by the *igu *mutant phenotype. The finding that motile cilia are largely unaffected in *igu *mutants, suggests that, in contrast to IFT proteins, DZIP1 function may be dedicated specifically to primary ciliogenesis.

## Methods

### Zebrafish strains and husbandry

Wild type embryos were obtained from AB or LWT strains. Mutant embryos were obtained from *igu*^*ts294e *^[19,20], *ptc1*^*hu1602*^*:ptc2*^*tj222 *^[25] and *smo*^*hi1640 *^[26]. Adult fish were maintained on a 14 h light/10 h dark cycle at 28°C in UK Home Office approved facilities in the Medical Research Council Centre for Developmental and Biomedical Genetics aquaria at the University of Sheffield.

### Isolation and tagging of the Gli2a BAC

Potential Gli2a-containing BACs were isolated using a bioinformatics approach, initially by blasting the cDNA sequence against all finished and unfinished sequences in the Sanger and Ensembl genome databases. The Spidey mRNA to genomic alignment program http://www.ncbi.nlm.nih.gov/spidey/ was then used to analyse exon presence and order in the sequence of potential BACs. The candidate BACs were then located in the Sanger tiling path and overlapping BACs on either side were also selected in order to cover as much as the Gli2a genomic region as possible. A total of seven BACs were obtained from RZPD (Berlin, Germany). Primers were designed to exons 1, 3, 6 and 13 of the Gli2a cDNA, and the presence of each of these exons in each of the BACs was tested by polymerase chain reaction (PCR) in order to identify a full-length clone. Genomic DNA was used as a positive control. CH211-216K4 was found to contain all of the Gli2a exons. BAC DNA was midi-prepped using the Nucleobond kit. Standard recombineering techniques [22] were used to generate a transgene encoding a C-terminally GFP-tagged form of Gli2a gene, replacing the stop codon of the Gli2a transcription unit with the start codon of the GFP coding region.

### Expression constructs

Sequencing of the full-length *GFP-igu *insert revealed that there were three amino acid substitutions that differed from the published *igu *cDNA sequence. These were at amino acid positions 65 (F>Y, TTT>TAT), 74 (V>A, GTG>GCG) and 488 (Q>H, CAG>CAT) of the published Genbank sequence.

Primers were designed to correct these errors by PCR:

"Correc65": CATCCCTCCTCCCT**T**TAAATTCAGATCCC

"Correc74": GGCGTGAAAATG**T**GGACTGGCGGCGC

"Correc488": ACAGGTCTTGGCA**G**AAGGAGGTGCAAG

The construct was then corrected using Stratagene's multi-site directed mutagenesis kit (Agilent Technologies, Cheshire, UK) according to the manufacturer's instructions and confirmed by sequencing. The full-length zebrafish tubulin-γ-complex-associated protein 3 (GTAP3) coding region was PCR amplified from embryonic reverse transcriptase-cDNA and cloned in frame with the ATG of tdTomato into the pCS2 vector.

A full-length zebrafish *foxj1a *was amplified by PCR and subcloned into pSGH2 vector [47] which contains 8xHeat shock elements and GFP reporter. The construct was injected into 1-cell stage embryos. The injected embryos were heat shocked at 11 hpf in the PCR machine; 22°C 30 min and 37°C 30 min, two cycles. The embryos were heat shocked again at 21 hpf and incubated at 28°C until fixation at 24 hpf.

### RNA *in situ *hybridization

*In situ *hybridization was performed essentially as previously described [48]. A partial *foxj1a *was isolated by PCR and a digoxigenin-labelled anti-sense *foxj1a *probe was prepared from a partial cDNA clone. The clone was linearized with NotI and transcribed with SP6 polymerase (Roche, Hertfordshire, UK).

### Immunohistochemistry

Immunohistochemistry was performed essentially as previously described [37]. Embryos were fixed in 4% paraformaldehyde in phosphate buffered saline. Mouse monoclonal anti-F59 (Hybridoma Bank) was used at 1:20 dilution and Mouse monoclonal anti-acetylated tubulin and anti-γ-tubulin (Sigma, Dorset, UK) were used at 1:200 dilution. Anti-mouse secondary antibody Cy3 (Jackson lab) was used at 1:200. Nuclei were labelled using a mounting medium with DAPI (Vectashield, CA, USA).

### *in vitro *mRNA transcription

Expression constructs were linearized with NotI and transcribed with SP6 polymerase. The SP6 mMessage Machine kit (Ambion, Texas, USA) was used for all *in vitro *transcriptions.

### Embryo injections

Wild type or mutant embryos were injected at the 1-cell stage with BAC DNA or capped messenger RNA using a Narishige IM-300 micromanipulators.

### Confocal microscopy

Immunolabelled embryos and live embryos (embedded in 1% low melting agarose) were analysed using an Olympus FV-1000 confocal microscope.

## Abbreviations

BAC: bacterial artificial chromosome; Ci: Cubitus interuptus; GFP: green fluorescent protein; Hh: Hedgehog; IFT: intraflagellar transport; *Igu*: Iguana; MFF: medial fast twitch muscle fibre; PCR: polymerase chain reaction; PKA: protein kinase A; Ptc: patched; Smo: smoothened; *yot*: *you too*.

## Authors' contributions

HRK participated in the experimental design, performed all of the Gli2-GFP localization studies and the *igu *mutant analysis and participated in drafting the manuscript. JR generated the BAC engineered Gli2-GFP fusion construct and corrected the *igu *cDNA sequence. FvE generated the *ptc1;ptc2 *double mutant animals and participated in the discussion of results. PWI conceived of the study, participated in the design and coordination of the experiments and drafted the manuscript. All authors read and approved the final manuscript.

## Supplementary Material

Additional file 1 **Supplementary Figure 1: Green fluorescent protein (GFP) does not localize to primary cilia.** In order to confirm that GFP alone does not localize to the primary cilia, we cloned GFP into pCS2 vector and injected into newly fertilised zebrafish eggs. Injected embryos were fixed at 18 hpf. Primary cilia and nuclei were visualised with acetylated tubulin (red) and DAPI (blue) respectively. GFP can be seen to localize to the cytoplasm and nuclei in both slow (b) and fast muscle (a) cells, but was not detected in the primary cilia. Green channels from (a) and (b) are shown in (c) and (d), respectively. Scale bar:10 μm Click here for file

Additional file 2 **Supplementary Figure 2. Gli2a-green fluorescent protein (GFP) positive cilia are longer than those lacking Gli2a-GFP protein.** We noticed that Gli2a-GFP positive cilia appear longer than other cilia, especially in *ptc1;ptc2 *double mutant embryos. In order to confirm this, we measured the length of the cilia in both Gli2a-GFP positive and negative cilia in *ptc1;ptc2 *double mutant and the difference in the mean lengths between the two groups was tested by the paired *t- *test. The difference was significant with *P *< 0.001 (a). In wild type embryos, the difference in the length of the cilia between Gli2a-GFP positive and Gli2a-GFP negative cilia was also significant, although not as great as in *ptc1;ptc2 *double mutant (b). We also compared the length of the Gli2a-GFP positive cilia in wild type and in *ptc1;ptc2 *double mutants, and again found a significant difference (c). The length of cilia lacking the Gli2a-GFP fusion protein, by contrast, did not differ between in wild type and *ptc1;ptc2 *mutant embryos. Click here for file
