# Supplementary figures and images for: Gli2a protein localization reveals a role for Iguana/DZIP1 in primary ciliogenesis and a dependence of Hedgehog signal transduction on primary cilia in the zebrafish
Source: BMC Biol. 2010 Apr 19;8:65. doi: 10.1186/1741-7007-8-65 (PMC2890509; doi:10.1186/1741-7007-8-65)

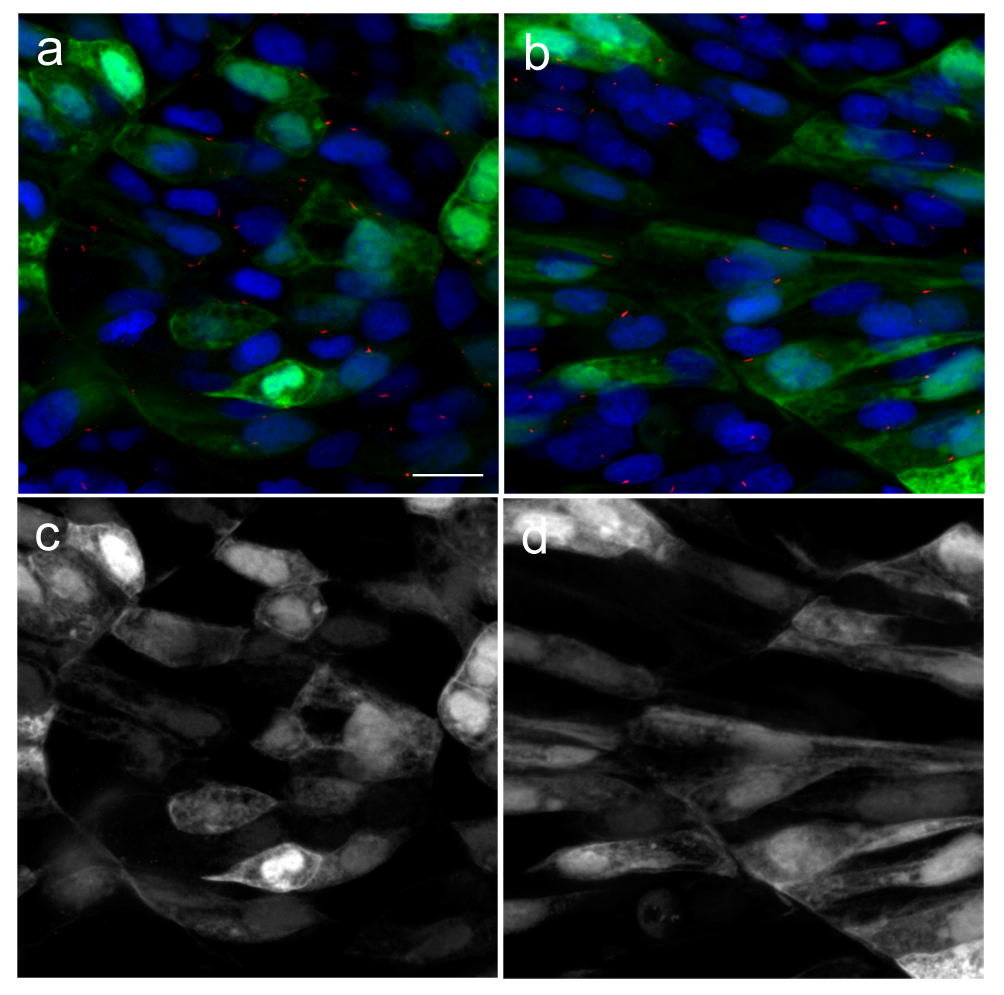

Supplement: Additional file 1 — Supplementary Figure 1: Green fluorescent protein (GFP) does not localize to primary cilia. In order to confirm that GFP alone does not localize to the primary cilia, we cloned GFP into pCS2 vector and injected into newly fertilised zebrafish eggs. Injected embryos were fixed at 18 hpf. Primary cilia and nuclei were visualised with acetylated tubulin (red) and DAPI (blue) respectively. GFP can be seen to localize to the cytoplasm and nuclei in both slow (b) and fast muscle (a) cells, but was not detected in the primary cilia. Green channels from (a) and (b) are shown in (c) and (d), respectively. Scale bar:10 μm [file 1741-7007-8-65-S1.tiff]

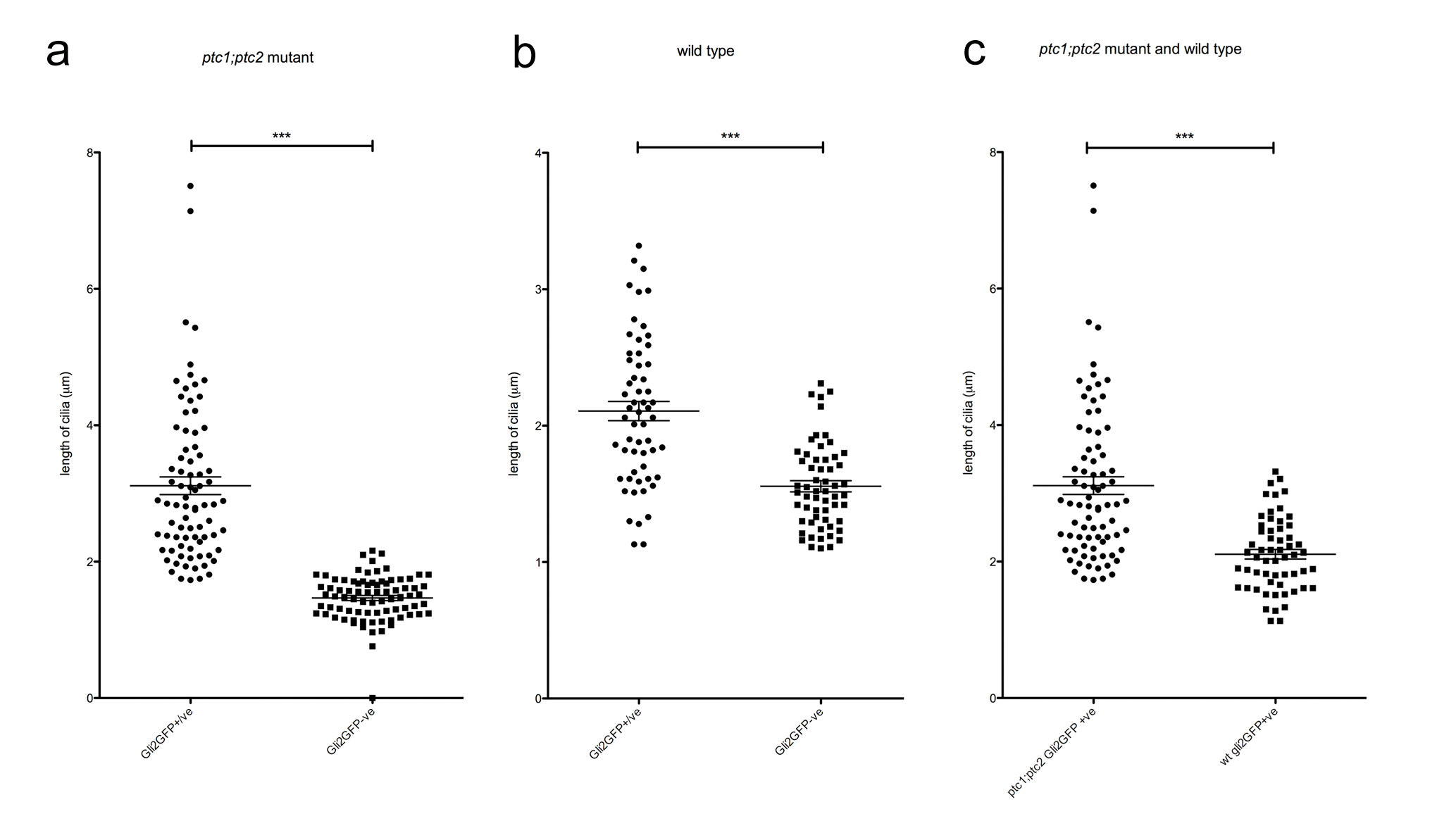

Supplement: Additional file 2 — Supplementary Figure 2. Gli2a-green fluorescent protein (GFP) positive cilia are longer than those lacking Gli2a-GFP protein. We noticed that Gli2a-GFP positive cilia appear longer than other cilia, especially in ptc1;ptc2 double mutant embryos. In order to confirm this, we measured the length of the cilia in both Gli2a-GFP positive and negative cilia in ptc1;ptc2 double mutant and the difference in the mean lengths between the two groups was tested by the paired t- test. The difference was significant with P < 0.001 (a). In wild type embryos, the difference in the length of the cilia between Gli2a-GFP positive and Gli2a-GFP negative cilia was also significant, although not as great as in ptc1;ptc2 double mutant (b). We also compared the length of the Gli2a-GFP positive cilia in wild type and in ptc1;ptc2 double mutants, and again found a significant difference (c). The length of cilia lacking the Gli2a-GFP fusion protein, by contrast, did not differ between in wild type and ptc1;ptc2 mutant embryos. [file 1741-7007-8-65-S2.tiff]
